# Achievement of Endoscopic Remission After Induction Reduces Hospitalization Burden in Crohn’s Disease: Findings From a Pooled Post Hoc Analysis of Risankizumab and Upadacitinib Phase III Trials

**DOI:** 10.1093/ecco-jcc/jjae128

**Published:** 2024-08-30

**Authors:** Remo Panaccione, Christopher Ma, Vipul Jairath, Axel Dignass, Namita Joshi, Ryan Clark, Jenny Griffith, Kristina Kligys, Monika Semwal, Zachary Smith, Dominic Mitchell, Dominic Nunag, Marc Ferrante

**Affiliations:** Division of Gastroenterology and Hepatology, University of Calgary, Calgary, AB, Canada; Division of Gastroenterology and Hepatology, University of Calgary, Calgary, AB, Canada; Division of Epidemiology and Biostatistics, Western University, London, ON, Canada; Division of Epidemiology and Biostatistics, Western University, London, ON, Canada; Alimentiv Inc., London, ON, Canada; Division of Gastroenterology, Schulich School of Medicine & Dentistry, Western University, London, ON, Canada; Department of Medicine I, Agaplesion Markus Hospital, Goethe University, Frankfurt Am Main, Germany; AbbVie Inc., North Chicago, IL, USA; AbbVie Inc., North Chicago, IL, USA; AbbVie Inc., North Chicago, IL, USA; AbbVie Inc., North Chicago, IL, USA; AbbVie Inc., North Chicago, IL, USA; Medicus Economics LLC, Boston, MA, USA; Medicus Economics LLC, Boston, MA, USA; Medicus Economics LLC, Boston, MA, USA; Department Gastroenterology and Hepatology, University Hospitals Leuven, KU Leuven, Leuven, Belgium

**Keywords:** Endoscopy, Crohn’s disease, hospitalization, mucosal healing

## Abstract

**Background:**

Endoscopic remission has emerged as an important treatment target in Crohn’s disease (CD) and has been associated with improvement in long-term outcomes. We examined the relationship between achievement of endoscopic remission and hospitalizations using pooled data from 52-week Phase III maintenance trials of risankizumab and upadacitinib in patients with moderate-to-severe active CD.

**Methods:**

Included patients received maintenance therapy after achieving a clinical response following a 12-week induction with risankizumab or upadacitinib. Endoscopic remission was defined as a Simple Endoscopic Score for Crohn’s Disease (SES-CD) of no greater than 4, with at least a 2-point reduction vs induction baseline and no subscore greater than 1. All subsequent hospitalization events were recorded until completion of the maintenance trial or discontinuation. Exposure-adjusted negative binomial regression models were estimated to assess the relationship between post-induction endoscopic remission and long-term hospitalization, controlling for demographics, clinical variables, and treatment arm.

**Results:**

Post-induction hospitalization rates were lower in patients who achieved endoscopic remission at the end of the induction period. In multivariable models, post-induction endoscopic remission was independently associated with incidence rate ratios of 0.45 (95% confidence interval [CI], 0.22-0.95, *p* = 0.036) and 0.71 (95% CI, 0.44-1.14, *p* = 0.156) for long-term disease-related and all-cause hospitalizations, respectively.

**Conclusions:**

Week 12 endoscopic remission is independently associated with a reduction in 52-week disease-related hospitalizations. However, achieving this stringent endpoint within 12 weeks of therapy may be challenging. Endoscopic response may be a more realistic early endoscopic target in the post-induction timeframe. Additional research is needed to evaluate early achievement of alternative endoscopic endpoints in CD.

## Graphical Abstract

**Graphical Abstract:**
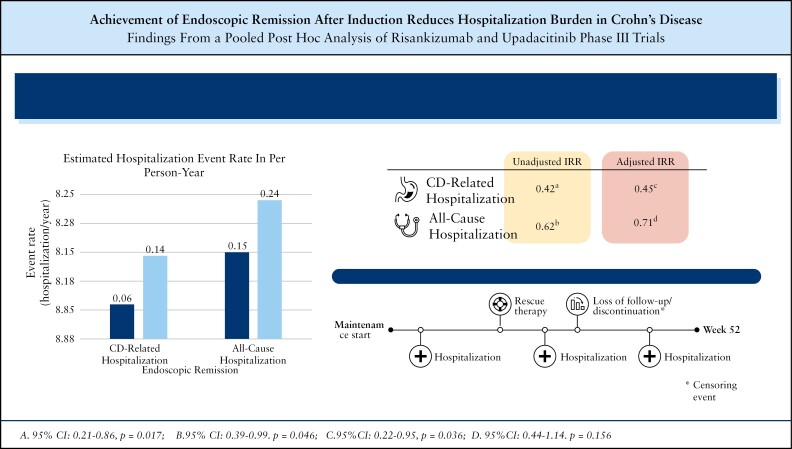

## 1. Introduction

Crohn’s disease (CD), a chronic inflammatory condition of the gastrointestinal tract, is characterized by heterogeneous symptoms including abdominal pain (AP), diarrhea, weight loss, and fatigue.^1^ Over time, this inflammation causes injury to the mucosal lining which may lead to stenosing and penetrating complications of the intestines.^2,3^ Endoscopy represents the gold standard for assessing the location, depth, and extent of mucosal lesions in the terminal ileum and colon.^4,5^ Its utility in diagnosis, monitoring, and surveillance in inflammatory bowel diseases (IBD) has been well established.^6^

Endoscopic remission is gaining recognition as a key treatment objective in the management of CD, to be considered alongside traditional measures in clinical trials such as the Crohn’s Disease Activity Index (CDAI),^7^ which captures the severity of near-term symptoms but may be poorly associated with findings of intestinal inflammation.^8–13^ As such, endoscopic endpoints have been included as co-primary or key secondary endpoints in recent CD clinical trials.^14–18^ The definition of endoscopic remission has evolved over time.^19^ However, endoscopic remission is most commonly defined as the absence of mucosal ulceration.^20,21^ The STRIDE-I and subsequent STRIDE-II IBD consensus, which reviewed treat-to-target strategies, identified endoscopic remission as the primary long-term target of therapy in CD.^22,23^ In draft guidance for the development of new drug therapies in CD, the US Food and Drug Administration (FDA) recommended that endoscopic remission be included as a co-primary endpoint in combination with clinical remission to be assessed at the end of the trial induction period and at Week 52 of the maintenance trial.^24^ (The draft guidance defines endoscopic remission as a Simple Endoscopic Score for Crohn’s Disease [SES-CD] of 0-2 or an SES-CD score of 0-4 with no subscore greater than 1, and clinical remission as a CDAI score of less than 150.)

Previous research has established a connection between endoscopically measured inflammation and the risk of hospitalization and surgery.^25,26^ A meta-analysis including retrospective and observational studies combined with post hoc analysis of randomized controlled trials found that endoscopic healing was associated with fewer hospitalizations and surgeries and predicted long-term clinical remission.^27^ Results from another meta-analysis indicated that endoscopic healing is associated with a greater likelihood of achieving long-term disease control/clinical remission with lower risks of hospitalization and surgery.^28^ More recently, results from the REACT-2 study showed that patients without active inflammation at baseline (C-reactive protein ≤ 5 mg/L) had fewer CD-related complications over a 2-year period.^29^ Whether early achievement of endoscopic remission changes the long-term natural history of CD is unclear. This study leverages data from the induction and 52-week Phase III post-induction maintenance trials for risankizumab and upadacitinib,^15,18^ the first CD therapies with FDA-approved labels citing endoscopic response and remission as a co-primary efficacy endpoint after both induction and post-induction maintenance.^30,31^ Using endoscopic data collected after induction therapy, this post hoc analysis tests the hypothesis that patients who have achieved early endoscopic remission have a lower rate of CD-related and all-cause hospitalizations events in the subsequent 52-week post-induction maintenance period.

## 2. Methods

### 2.1. Study design and patients

This study is based on a post hoc analysis of pooled data from 2 Phase III, multicenter, double-blind, placebo-controlled, randomized trials for upadacitinib (U-ENDURE, NCT03345823) and risankizumab (FORTIFY, NCT03105102), described in detail elsewhere.^15,18^ These 52-week post-induction maintenance trials included patients who achieved a clinical response after a 12-week induction period with an active therapy (risankizumab or upadacitinib). Clinical response was defined as a ≥30% decrease in average daily stool frequency (SF) and/or a ≥30% decrease in average daily AP score with no worsening from baseline in either measure.

In FORTIFY, 12-week clinical responders to intravenous (IV) risankizumab in the ADVANCE (NCT03105128) or MOTIVATE (NCT03104413) induction studies were randomly assigned (1:1:1) to receive either subcutaneous (SC) risankizumab 180 mg, SC risankizumab 360 mg, or SC placebo every 8 weeks (Q8W).^18^ The 12-week clinical responders were categorized as follows:

- First induction responders: Week 12 clinical responders to IV risankizumab (600 or 1200 mg at Weeks 0, 4, and 8).
- Second induction responders: First induction placebo patients with inadequate Week 12 response who responded to IV risankizumab (1200 mg at Weeks 12, 16, and 20) at Week 24.

Patients who received a 24-week prolonged induction treatment with risankizumab were excluded from this study. Included patients were aged 16-80 years with moderate-to-severe active CD defined as a CDAI of 220-450, mean daily SF of at least 4 or mean AP score of at least 2, and an SES-CD of ≥6 (or ≥4 for isolated ileal disease) at baseline of the induction studies. An ileocolonoscopy was performed both at baseline and at the Week 52/Premature Discontinuation (PD) visit for the post-induction maintenance trial and was evaluated by a central reader.

In U-ENDURE, 12-week clinical responders to upadacitinib 45 mg once daily (QD) in the U-EXCEL (NCT03345849) and U-EXCEED (NCT03345836) induction studies were randomized to receive 15 mg of upadacitinib, 30 mg of upadacitinib, or placebo (1:1:1 ratio) QD.^15^ The 12-week clinical responders were categorized as follows:

- First induction responders: Week 12 clinical responders to upadacitinib 45 mg QD.
- Second induction responders: First induction placebo patients with inadequate Week 12 response who responded to upadacitinib 45 mg QD at Week 24.

Patients who received a 24-week prolonged induction treatment with upadacitinib were excluded from this study. At baseline, patients were aged 18-75 years and had moderate-to-severe CD for at least 3 months. Moderate-to-severe CD was defined as an average of 4 or more instances of very soft or liquid stools daily or an AP score of 2 or more, plus an SES-CD of ≥6 (≥4 for patients with isolated ileal disease), excluding the narrowing component of the scale.^15^ As the eligibility criteria differed from that in the risankizumab induction trials, only the subset of patients in U-ENDURE with induction baseline CDAI scores of 220-450 were included in the pooled dataset. As in FORTIFY, a centrally read ileocolonoscopy was performed both at baseline and at Week 52/PD of the post-induction maintenance phase to assess endoscopic outcomes.

In both trials, patients had a history of failure of one or more conventional or biologic therapies.^15,18^ Concomitant use of biologic therapies and immunosuppressants other than methotrexate or glucocorticoids was not permitted. In addition, the FORTIFY study allowed continued treatment with azathioprine and 6-mercaptopurine in patients who were receiving stable doses at Week 0. Patients who were initially receiving glucocorticoids began a protocol-specified taper.

### 2.2. Clinical outcomes

The primary objective was to examine the relationship between endoscopic remission after an induction period and subsequent CD-related and all-cause hospitalizations. Post-induction maintenance-baseline endoscopic remission was determined using SES-CD scores calculated from the last endoscopic assessment in an induction-phase trial. These assessments were performed 12 weeks from the start of active treatment (at Week 12 for patients randomized to an active treatment arm and Week 24 for placebo patients who responded to an active treatment during the second induction period).

**Figure 1:**
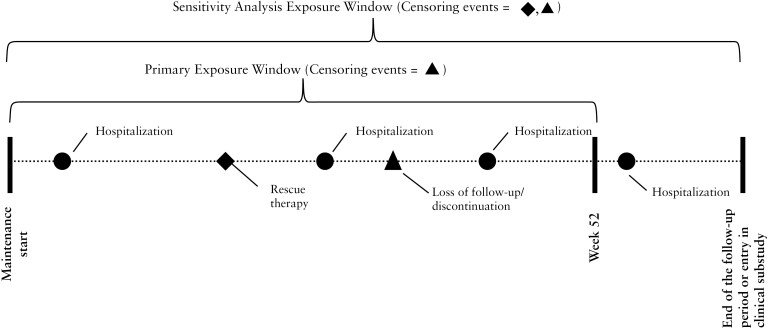
Primary and sensitivity exposure windows for counting hospitalization events. Figure 1 depicts the exposure windows and censoring events considered in the primary and sensitivity analyses. The sensitivity analysis exposure window included hospitalization events up to the end of the follow-up period or the entry in a post-maintenance clinical study, but censored events occurring after initiation of rescue therapy as the patients’ time-at-risk stopped at initiation of rescue therapy.

Endoscopic remission status was determined according to the trial methodology for assessing achievement of this endpoint in induction-phase efficacy analyses. Patients were considered to be in endoscopic remission if they had an SES-CD of ≤4 with at least a 2-point reduction vs baseline and no subscore >1 in any individual variable, as scored by a central reviewer. Following trial conventions, the 34 patients without available endoscopic data post-induction were included in the nonremission group. For each patient, all unique all-cause and CD-related hospitalization events were counted within 2 time-at-risk periods: a primary exposure window and a sensitivity analysis exposure window. Figure 1 illustrates the differences between these windows.

The relationship between endoscopic response after an induction period and subsequent CD-related and all-cause hospitalizations was explored. These analyses are presented in the Supplementary Material.

#### 2.2.1. Definition of the time-at-risk for the primary exposure window

Hospitalization events counted in the primary exposure window were those occurring after receipt of the first post-induction maintenance study dose (risankizumab, upadacitinib, or placebo) up to the earliest of: (a) the Week 52 (end-of-trial) endoscopic assessment (or 52 weeks from the first study dose if no Week 52 endoscopic data were collected) or (b) study discontinuation/loss of follow-up. The definition of the sensitivity analysis time-at-risk window is presented in Section A.1 of the Supplementary Material.

### 2.3. Statistical analysis

Analyses were performed in the intent-to-treat population used to evaluate primary endpoints in the post-induction maintenance trials; included patients were randomized and received 1 dose of the study drug. Baseline characteristics of the population were tabulated, with frequencies and percentages summarizing categorical variables and means and SDs describing continuous measures. Statistical differences in baseline covariates between patients in and out of endoscopic remission were assessed using chi-squared tests for categorical variables and Wilcoxon rank-sum tests for continuous variables.

For patients in each group, hospitalization rates were calculated as the number of events divided by the number of patient-years of observation. Incidence rate ratios (IRRs) comparing hospitalization rates for those achieving endoscopic remission vs those failing to achieve endoscopic remission, as well as 95% confidence intervals (CIs), were estimated using exposure-adjusted univariable and multivariable negative binomial regression models. Multivariable models used data from the subset of patients with all available observations and controlled for patient demographics (age, sex, race [White, Asian, other], ethnicity [Hispanic vs non-Hispanic]), pre-induction (baseline) steroid use (yes/no), previous failure (inadequate response) of a biologic/anti-tumor necrosis factor therapy (yes/no), disease duration at induction baseline, disease phenotype (colonic-only, ileal-only, ileal-colonic) as assessed by pre-induction endoscopy, and post-induction maintenance-baseline CDAI. In all analyses, a 2-sided alpha error level of 0.05 was used to indicate statistical significance. All analyses were conducted using SAS 9.4 software (SAS Institute Inc., Cary, NC, USA).

### 2.4. Ethical considerations

All 6 randomized controlled trials were conducted in compliance with the respective study protocols, the International Conference on Harmonization guidelines, and the ethical principles of the Declaration of Helsinki. As per Good Clinical Practice, the study protocol, informed consent forms, and all other explanatory materials were approved by the relevant ethics committees or institutional review boards at all study sites. All patients provided informed consent before study participation.

## 3. Results

### 3.1. Study population

The pooled dataset consisted of 462 patients from FORTIFY and 407 patients from U-ENDURE who met the induction baseline CDAI criterion of 220-450. As described in Section 2.1, this criterion was imposed to ensure that all included patients had comparable disease severity at baseline; 95 patients from the U-ENDURE study with baseline CDAI outside of this range were excluded. The mean patient exposure time across all 869 patients included in the analysis was 344.4 days (SD: 63.5).

Baseline characteristics of the studied population are presented in Table 1. Overall, 235 patients (27%) had endoscopic remission at post-induction maintenance-baseline. The mean age of the population was 38 years, 54.2% were male, and the majority were White (75%); other demographics were similar across patients with or without endoscopic remission. Compared with the endoscopic nonremission group, those with endoscopic remission were less likely to have ileal-only disease (8% vs 14%, *p* = 0.021), less likely to have previously failed a biologic therapy (64% vs 77%, *p* < 0.001), and had shorter average disease duration (8.5 vs 10.5 years, *p* < 0.001). The mean CDAI score at post-induction maintenance-baseline was also lower among patients in endoscopic remission compared with those without endoscopic remission (122.7 vs 144.5, *p* < 0.001). The post-induction SES-CD score was 1.2 in the endoscopic remission group and 10.6 in the endoscopic nonremission group.

**Table 1:** Patient demographics and post-induction maintenance-baseline characteristics.

		Endoscopic remission		
Parameter	Overall sample [*n* = 869]	Yes [*n* = 235]	No [*n* = 634]	*p* Value
Age in years, mean (SD)	37.9 (13.4)	37.4 (12.8)	38.1 (13.6)	0.620
Male, *n* (%)	471 (54.2)	119 (51.0)	352 (56.0)	0.200
Race, *n* (%)				
Asian	168 (19.3)	47 (20.0)	121 (19.0)	0.762
Other	50 (5.8)	14 (6.0)	36 (6.0)	0.875
White	651 (74.9)	174 (74.0)	477 (75.0)	0.718
Ethnicity, *n* (%)				
Hispanic	51 (5.9)	17 (7.0)	34 (5.0)	0.297
Non-Hispanic	818 (94.1)	218 (93.0)	600 (95.0)	
Disease location, *n* (%)				
Ileal-only	107 (12.3)	19 (8.0)	88 (14.0)	0.021
Colonic-only	353 (40.6)	110 (47.0)	243 (38.0)	0.024
Ileal-colonic	409 (47.1)	106 (45.0)	303 (48.0)	0.481
Disease duration in years, mean (SD)	10.0 (9.0)	8.5 (8.8)	10.5 (9.0)	<0.001
Pre-induction baseline steroid use, *n* (%)	300 (34.5)	83 (35.0)	217 (34.0)	0.764
Previous biologic failure, *n* (%)	642 (73.9)	151 (64.0)	491 (77.0)	<0.001
Maintenance treatment arm, *n* (%)^a^				
Placebo (RZB trial)^a^	164 (18.9)	46 (20.0)	118 (19.0)	0.747
Placebo (UPA trial)^a^	130 (15.0)	33 (14.0)	97 (15.0)	0.644
Risankizumab 180 mg SC Q8W	157 (18.1)	44 (19.0)	113 (18.0)	0.759
Risankizumab 360 mg SC Q8W	141 (16.2)	39 (17.0)	102 (16.0)	0.857
Upadacitinib 15 mg QD	131 (15.1)	38 (16.0)	93 (15.0)	0.583
Upadacitinib 30 mg QD	146 (16.8)	35 (15.0)	111 (18.0)	0.360
CDAI, mean (SD)	138.6 (74.0)	122.7 (73.7)	144.5 (73.3)	<0.001
SES-CD, mean (SD)	8.0 (6.9)	1.2 (1.5)	10.6 (6.4)	<0.001

Abbreviations: CDAI, Crohn’s Disease Activity Index; Q8W, once every 8 wk; QD, once daily; RZB, risankizumab; SC, subcutaneous; SES-CD, Simple Endoscopic Score for Crohn’s Disease; UPA, upadacitinib.

^a^These are the treatment arms resulting from the re-randomization before the entry in the 52-wk post-induction maintenance studies not to be confounded with the induction studies randomization arms. Patients randomized to placebo for the post-induction maintenance study are presented separately based on the post-induction maintenance Phase III study source (RZB or UPA trial).

### 3.2. Association of endoscopic remission and CD-related hospitalizations

**Figure 2:**
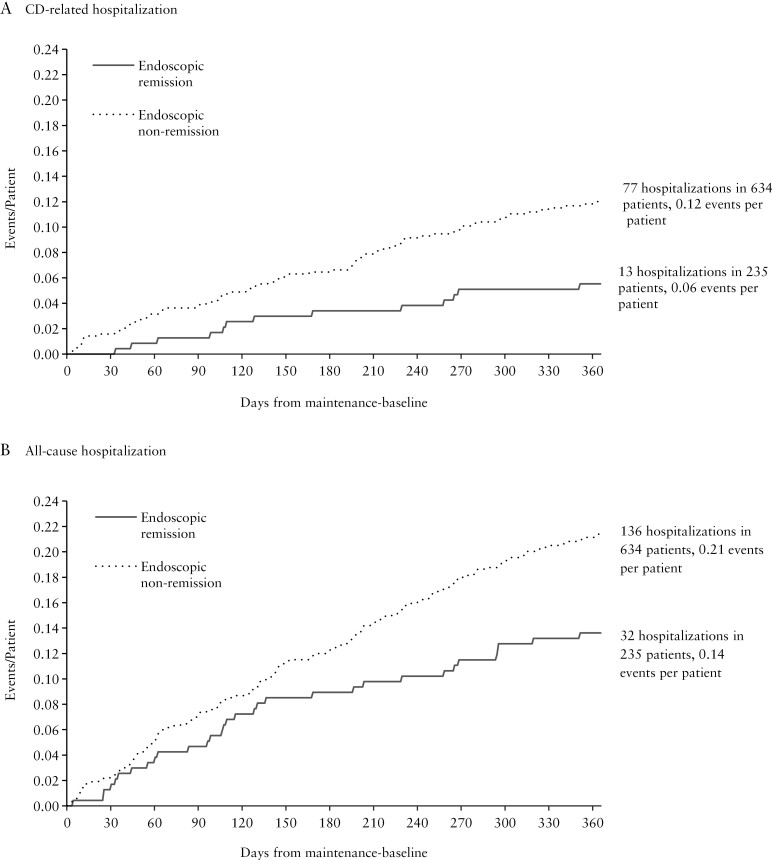
Cumulative number of hospitalization events per patient over time stratified by endoscopic remission status in the primary exposure window. Abbreviation: CD, Crohn’s disease.

A total of 90 distinct CD-related hospitalizations were recorded during the primary exposure period (post-induction maintenance-baseline to Week 52 or loss of follow-up). The timing of CD-related hospitalization was relatively uniform across the study period, with approximately 25% of events occurring within the first 62 days, and approximately 75% of events occurring within the first 242 days. Of recorded events, 77 CD-related hospitalizations occurred in the 634 nonremission patients in a cumulative exposure time of 595.3 person-years (0.129 events per person-year). By contrast, among the 235 patients with endoscopic remission, only 13 CD-related hospitalizations were recorded over a total exposure time of 224.0 person-years (0.058 events per person-year). Cumulative CD-related hospitalization events by time from study baseline for each group are shown in Figure 2A. Similar results were obtained when using the sensitivity analysis exposure window presented in the Supplementary Figure 1.

**Figure 3:**
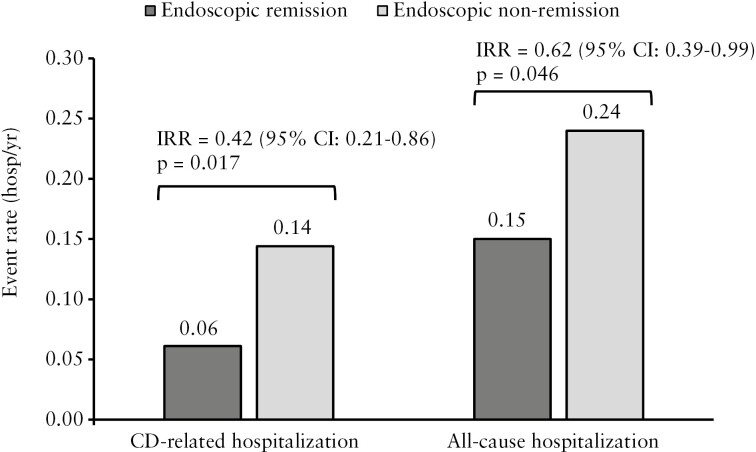
Predicted hospitalization rates by endoscopic remission status in unadjusted models. Abbreviations: CD, Crohn’s disease; CI, confidence interval; hosp, hospitalizations; IRR, incidence rate ratio; yr, year.

In negative binomial regression models including only remission status (yes vs no), the independent effect of endoscopic remission at post-induction maintenance-baseline was associated with a statistically significant reduction in CD-related hospitalizations (IRR = 0.42, 95% CI, 0.21-0.86, *p* = 0.017), corresponding to a 58% reduction in the predicted annual event rate (0.06 vs 0.14 events per person-year, Figure 3). Results in multivariable models (Table 2) were similar in magnitude: Endoscopic remission was independently associated with a 55% reduction in CD-related hospitalization rate (IRR = 0.45, 95% CI, 0.22-0.95, *p* = 0.036), regardless of the post-induction maintenance treatment arm.

**Table 2:** Multivariable negative binomial regression results for Crohn’s disease-related hospitalization rates.

Parameter	IRR (95% CI)	Coefficient (SE)	*p* Value
Endoscopic remission	0.45 (0.22-0.95)	−0.792 (0.377)	0.036
Age (+5 y)	0.95 (0.84-1.08)	−0.050 (0.063)	0.426
Female	1.25 (0.72-2.17)	0.226 (0.281)	0.420
Race (vs White)			
Asian	1.95 (1.01-3.77)	0.670 (0.335)	0.045
Other	0.98 (0.27-3.59)	−0.025 (0.664)	0.970
Hispanic (vs not)	1.79 (0.64-4.96)	0.580 (0.521)	0.266
Disease location (vs colonic)			
Ileal-only	2.69 (1.03-7.01)	0.990 (0.489)	0.043
Ileal-colonic	1.91 (1.05-3.49)	0.649 (0.307)	0.034
Disease duration (years)	0.99 (0.95-1.03)	−0.008 (0.020)	0.672
Pre-induction (baseline) steroid use	2.58 (1.49-4.47)	0.948 (0.280)	<0.001
Previous biologic failure	2.71 (1.22-6.02)	0.996 (0.408)	0.015
CDAI (+10, maintenance-baseline)	1.03 (0.99-1.06)	0.025 (0.019)	0.200
Post-induction maintenance treatment (vs placebo [UPA trial])^a^			
Placebo (RZB trial)^a^	0.32 (0.13-0.78)	−1.134 (0.451)	0.012
RZB 180 mg SC Q8W	0.19 (0.07-0.54)	−1.650 (0.525)	0.002
RZB 360 mg SC Q8W	0.40 (0.16-0.99)	−0.920 (0.465)	0.048
UPA 15 mg QD	0.65 (0.27-1.52)	−0.438 (0.437)	0.316
UPA 30 mg QD	0.43 (0.18-1.01)	−0.853 (0.442)	0.054

Abbreviations: CDAI, Crohn’s Disease Activity Index; CI, confidence interval; IRR, incidence rate ratio; Q8W, once every 8 wk; QD, once daily; RZB, risankizumab; SC, subcutaneous; UPA, upadacitinib.

^a^These are the treatment arms resulting from the re-randomization before the entry in the 52-week post-induction maintenance studies not to be confounded with the induction studies randomization arms. Patients randomized to placebo for the post-induction maintenance study are presented separately based on the post-induction maintenance Phase III study source (RZB or UPA trial).

Previous failure of a biologic therapy (IRR = 2.71, 95% CI, 1.22-6.02, *p* = 0.015) and pre-induction (baseline) use of steroids (IRR = 2.58, 95% CI, 1.49-4.47, *p* < 0.001) were also associated with more CD-related hospitalizations. There were significant differences in CD-related hospitalization rates among patients with ileal-only disease (IRR = 2.69, 95% CI, 1.03-7.01, *p* = 0.043) and ileal-colonic disease (IRR = 1.91, 95% CI, 1.05-3.49, *p* = 0.034) compared to those with isolated colonic disease. The model also estimated higher rates of CD-related hospitalizations for Asians vs White patients (IRR = 1.95, 95% CI, 1.01-3.77, *p* = 0.045).

Section A.3 of the Supplementary Material presents the multivariable models for Week 12 endoscopic response and 52-week CD-related hospitalizations.

### 3.3. Association of endoscopic remission and all-cause hospitalization

A total of 168 distinct all-cause hospitalization events (including CD-related hospitalizations) were recorded during the primary exposure period. Hospitalizations occurred relatively uniformly, with approximately 25% of events occurring within the first 61 days of the trial period and approximately 75% of events occurring within the first 240 days. As with CD-related hospitalization, most events occurred within the non-endoscopic remission cohort where 136 all-cause hospitalizations were recorded among the 634 patients, corresponding to an event rate of 0.228 per person-year. For those in endoscopic remission, 32 all-cause hospitalizations occurred, translating to 0.143 events per person-year. Cumulative events over the study period for patients in and out of remission are shown in Figure 2.

Univariable models estimated a statistically significant decrease in all-cause hospitalization associated with endoscopic remission (IRR = 0.62, 95% CI, 0.39-0.99, *p* = 0.046), with predicted all-cause hospitalizations rates of 0.15 and 0.24 events per person-year among patients meeting and failing to meet this endpoint, respectively (Figure 3). As shown in Table 3, a decreased hospitalization burden was independently associated with endoscopic remission after adjusting for covariates, although results were no longer statistically significant (IRR = 0.71, 95% CI, 0.44-1.14, *p* = 0.156). Other significant predictors of all-cause hospitalization included the use of steroids in the pre-induction (baseline) period (IRR = 2.02, 95% CI, 1.35-3.03, *p *< 0.001), Asian vs White race (IRR = 1.77, 95% CI, 1.08-2.90, *p* = 0.024), age (IRR = 1.08 per 5-year increase, *p* = 0.048), and post-induction maintenance-baseline CDAI (IRR = 1.03 per 10-unit increase, 95% CI, 1.01-1.06, *p* = 0.010). Sensitivity analysis results are presented in Section A.5 of the Supplementary Material.

**Table 3:** Multivariable negative binomial regression results for all-cause hospitalization rates.

Parameter	IRR (95% CI)	Coefficient (SE)	*p* Value
Endoscopic remission	0.71 (0.44-1.14)	−0.346 (0.243)	0.156
Age (+5 y)	1.08 (1.00-1.17)	0.079 (0.040)	0.048
Female	1.23 (0.83-1.82)	0.205 (0.200)	0.306
Race (vs White)			
Asian	1.77 (1.08-2.90)	0.570 (0.253)	0.024
Other	0.99 (0.39-2.48)	−0.013 (0.471)	0.979
Hispanic (vs not)	1.29 (0.58-2.86)	0.254 (0.406)	0.531
Disease location (vs colonic)			
Ileal-only	1.47 (0.77-2.83)	0.388 (0.333)	0.244
Ileal-colonic	1.48 (0.97-2.26)	0.390 (0.217)	0.072
Disease duration (years)	0.99 (0.97-1.02)	−0.008 (0.012)	0.503
Pre-induction (baseline) steroid use	2.02 (1.35-3.03)	0.704 (0.206)	<0.001
Previous biologic failure	1.47 (0.90-2.39)	0.383 (0.249)	0.124
CDAI (+10, maintenance-baseline)	1.03 (1.01-1.06)	0.034 (0.013)	0.010
Post-induction maintenance treatment (vs placebo [UPA trial])^a^			
Placebo (RZB trial)^a^	0.44 (0.23-0.82)	−0.826 (0.321)	0.010
RZB 180 mg SC Q8W	0.42 (0.22-0.81)	−0.856 (0.331)	0.010
RZB 360 mg SC Q8W	0.53 (0.27-1.01)	−0.644 (0.332)	0.052
UPA 15 mg QD	0.50 (0.26-0.99)	−0.686 (0.343)	0.045
UPA 30 mg QD	0.56 (0.30-1.06)	−0.574 (0.321)	0.074

Abbreviations: CDAI, Crohn’s Disease Activity Index; CI, confidence interval; IRR, incidence rate ratio; Q8W, once every 8 wk; QD, once daily; RZB, risankizumab; SC, subcutaneous; UPA, upadacitinib.

^a^These are the treatment arms resulting from the re-randomization before the entry in the 52-wk post-induction maintenance studies not to be confounded with the induction studies randomization arms. Patients randomized to placebo for the post-induction maintenance study are presented separately based on the post-induction maintenance Phase III study source (RZB or UPA trial).

Section A.4 of the Supplementary Material presents the multivariable models for Week 12 endoscopic response and 52-week all-cause hospitalizations.

## 4. Discussion

This study contributes to a growing body of research linking endoscopic outcomes with a reduced risk of severe disease-related complications in CD. Our analysis used data from randomized controlled trials to assess the impact of endoscopic remission on the reduction of CD-related complications in a 1-year follow-up period in patients who achieved clinical response (see Section 2.1) after a 12-week induction therapy. We found that early endoscopic remission status, as determined by SES-CD total score at the end of induction therapy, was associated with a reduction in the subsequent rate of hospitalizations in models controlling for contemporaneous measures of disease severity (CDAI) as well other clinical and demographic characteristics. The estimated all-cause hospitalization rate reduction was similar in size but not statistically significant, potentially because of a weaker connection between endoscopic outcomes and other hospitalization events.

We have also explored the association between endoscopic response and hospitalizations. The point estimates suggest a potential protective effect from achieving endoscopic response on all-cause and CD-related hospitalizations. However, the results did not reach statistical significance. The estimated IRRs for all-cause and CD-related hospitalizations were 0.97 (95% CI, 0.64-1.46) and 0.77 (95% CI, 0.43-1.39), respectively. There are multiple reasons that could explain why the endoscopic response results were not significant. The post hoc study excludes patients who did not meet the clinical response criteria of the induction trials, defined as a ≥30% decrease in average daily SF and/or a ≥30% decrease in average daily AP score (both not worse than baseline at Week 12). While the post hoc study only shows statistical significance with the stricter endoscopic remission, the exclusion of patients who failed to achieve clinical response has a negative impact on measures like endoscopic response. Although the protective association of endoscopic healing with improved long-term outcomes in CD has been previously demonstrated, achieving this stringent endpoint within 12 weeks of therapy may be challenging. Thus, even though the protective effect for endoscopic response did not reach statistical significance, in part due to the study design, endoscopic response may be more realistic outcome for clinicians in the post-induction timeframe.

Other predictors of hospitalization were prior biologic failure and pre-induction baseline steroid use, which can indicate refractory disease less likely to respond to treatment. Relative to colonic-only disease, ileal-only and ileal-colonic disease were also associated with more hospitalizations. This result is expected as ileal disease is more commonly associated with (subclinical) complications, which, over time, may lead to hospitalization and surgery. For instance, Golovics et al^32^ found that colonic disease was associated with fewer major IBD-related abdominal surgery (odds ratio = 0.33; *p* = 0.002). There was also evidence of race being a predictor, with Asian patients more likely to be hospitalized than White patients. There is mixed evidence that race is associated with differential outcomes in CD, with some research finding worse outcomes in White patients^33,34^ and others reporting higher hospitalization rates among non-White populations.^35,36^ As in these studies, race was not the primary variable of interest in our analysis and there are many unobserved characteristics including study-site-specific geographic, cultural, and socioeconomic factors which could contribute to this effect.^35^ Post-induction maintenance-baseline CDAI was not a significant predictor of hospitalization rate, although consideration of only induction-phase responders limited the range of measurements compared to a general patient population. Post-induction treatment arm IRR should be interpreted with caution in the context of this pooled post hoc study. The inclusion of treatment arm covariates in the negative binomial regression should be viewed as a control for potential remaining differences between the 2 Phase III clinical trials, thus the placebo arms were kept study specific. Adding categorical geographical region variables in the multivariable analyses to account for potential regional differences in hospitalization rates did not weaken the association between endoscopic outcomes and hospitalization rates.

Trial data represent a rich source for studying the clinical value of endoscopic remission as standardized, carefully measured endoscopic values are typically collected.^5,37^ The risankizumab and upadacitinib clinical trials co-primary endpoints (clinical response and endoscopic remission) required collecting early endoscopic data during the induction studies and at Week 52 of the maintenance studies. Prospective studies leveraged in prior research may not have had sufficient sample sizes to identify statistically significant hospitalization effects.^17,27,28^ However, the results in this study agree with those published in a combined source meta-analysis, which found that complete endoscopic healing provides a protective effect (relative risk [RR] < 1) against hospitalizations (RR = 0.47; 95% CI, 0.33-0.67) and surgeries (RR = 0.39; 95% CI, 0.25-0.60), when comparing to patients without endoscopic healing.^20,27^

Patients with CD experience reduced quality of life and bear significant economic costs, including those attributable to an elevated risk of hospitalization.^38–41^ Up to 80% of patients with CD will require hospitalization.^8^ A 2020 retrospective study of 6715 patients with CD reported an average of 0.34 inpatient admissions per year with an associated per patient per year (PPPY) direct medical cost of $24 500 (2017 USD).^41^ Annual direct medical costs (PPPY) were $44 934 in the moderate-to-severe CD subgroup (*N* = 3890) and $101 013 in the CD-related surgery subgroup (*N* = 690).^41^ Hospitalization costs comprise a substantial fraction of the lifetime medical costs incurred by the typical patient—estimated to be $622 056 in 2016 USD—of which $164 298 are incurred in the inpatient setting.^41,42^

Our approach to this study was to analyze the impact of endoscopic remission from a treatment agnostic perspective. Including treatment arm covariates with specific placebo arms for risankizumab and upadacitinib allowed us to control for differences across studies. The multivariable regression results presented in Tables 2 and 3 and Supplementary Table 1 suggest that compared to upadacitinib maintenance study, there is a statistically significant protective carry over effect associated with the risankizumab maintenance study. This observation is coherent with the study Week 12 induction trial results^15,16^ and the Week 52 maintenance results^15,18^ (see Supplementary Tables 2–5) when looking at CDAI clinical remission, endoscopic response, and endoscopic remission. While the weighted average Week 12 results are comparable between the 2 studies, the risankizumab study performed relatively well compared to the upadacitinib study as reflected by the Week 52 results. This explains the large significant protective effect observed in the post hoc study results for the risankizumab placebo arm.

This study has several limitations. Although the upadacitinib and risankizumab clinical programs shared similar study protocols, there are inherent differences between the 2 interventions. Risankizumab is an injection drug with a mean terminal half-life of 21-28 days,^43^ while upadacitinib is a daily oral drug with a mean terminal half-life of 8-14 hours.^44^ This point led to differences with regard to the steroid tapering protocols. For risankizumab, the corticosteroid tapering protocol was initiated only at the maintenance study Week 0, while for upadacitinib, the corticosteroid tapering protocol was initiated at Week 4 of the induction studies and continued during the maintenance study.^15,16,18^

Overall, on average, patients were at risk of hospitalization during 94% of the 52-week follow-up period (49 weeks). The average time-at-risk was slightly higher for risankizumab (98.3% for patients without endoscopic remission and 99.6% for patients with endoscopic remission) than for upadacitinib patients (89.0% for patients without endoscopic remission and 90.1% for patients with endoscopic remission). This difference in follow-up may be related to differences in the mode of administration of the 2 drugs and their dosing schedule. The maintenance risankizumab dosing schedule is 360 mg by SC injection Q8W, whereas upadacitinib maintenance treatment corresponds to 15 or 30 mg daily oral administration.

Data were limited to patients who experienced a clinical response at the end of the induction-phase trials; nonresponders were not eligible for inclusion in the post-induction maintenance trial and were lost to follow up. Members of the latter group were potentially more likely to require hospitalization, but the effect of endoscopic remission or endoscopic response status on clinical complications could not be assessed in this population. The exclusion of patients who had not satisfied the end of induction clinical response most likely weakened the association between endoscopic and hospitalization outcomes. In this setting, a stricter measure like endoscopic remission, as opposed to a relative measure such as endoscopic response, will be advantaged. Therefore, the nonstatistical significance of endoscopic response on hospitalization should be interpreted with caution. In addition, as this analysis was conducted post hoc, sample sizes were potentially underpowered to detect predictors of hospitalization rate, given the rarity of these events.

The achievement of endoscopic remission is an important goal in treatment of CD. However, monitoring mucosal healing through endoscopic procedures has practical implications.^20^ Endoscopy is invasive and only allows visualization of the superficial layer of the colon and terminal ileum. Noninvasive imaging and biomarkers are still being assessed as reliable means for assessing endoscopic endpoints. Despite these limitations, endoscopic remission is an increasingly important treatment goal for patients with CD, with greater emphasis placed on the presence and degree of mucosal lesions in clinical trials, treat-to-target recommendations, and regulatory guidance.^14–16,22–25^ While endoscopic outcomes have typically been considered long-term target, the current study suggests that achievement of early endoscopic remission may reduce subsequent disease-related complications.

## 5. Conclusion

This post hoc study leverages high-quality centrally read endoscopic measurements and detailed tracking of both the timing and cause of patient hospitalizations. The study results indicate that achieving endoscopic remission after 12-week induction therapy was independently associated with a significant reduction in CD-related hospitalizations through the 52-week post-induction maintenance period among patients who met the clinical response criteria of the induction studies. This study adds to a growing body of literature suggesting that reductions in mucosal inflammation may alter disease trajectory, reducing the clinical and economic burden associated with CD over time. Future studies should explore whether the early achievement of endoscopic endpoints adds additional benefits over achieving these endpoints at later time points.

## Supplementary Material

jjae128_suppl_Supplementary_Materials

## Data Availability

AbbVie is committed to responsible data sharing regarding the clinical trials we sponsor. This includes access to anonymized, individual, and trial-level data (analysis data sets), as well as other information (eg, protocols, clinical study reports, or analysis plans), as long as the trials are not part of an ongoing or planned regulatory submission. This includes requests for clinical trial data for unlicensed products and indications. These clinical trial data can be requested by any qualified researchers, who engage in rigorous, independent, scientific research, and will be provided following review and approval of a research proposal, Statistical Analysis Plan (SAP), and execution of a Data Sharing Agreement (DSA). Data requests can be submitted at any time after approval in the United States and Europe and after acceptance of this manuscript for publication. The data will be accessible for 12 months, with possible extensions considered. For more information on the process or to submit a request, visit the following link: https://vivli.org/ourmember/abbvie/ and then select “Home.”
