# Supplementary material for: Achievement of Endoscopic Remission After Induction Reduces Hospitalization Burden in Crohn’s Disease: Findings From a Pooled Post Hoc Analysis of Risankizumab and Upadacitinib Phase III Trials
Source: J Crohns Colitis. 2024 Aug 30;19(2):jjae128. doi: 10.1093/ecco-jcc/jjae128 (PMC11836885; doi:10.1093/ecco-jcc/jjae128)
Supplement: jjae128_suppl_Supplementary_Materials [file jjae128_suppl_supplementary_materials.docx]

- 1. Definition of the time-at-risk for the sensitivity analysis exposure window

Hospitalization events counted in the sensitivity analysis exposure window were those occurring after receipt of the first post-induction maintenance dose (of risankizumab, upadacitinib, or placebo) up to the earliest of (a) study discontinuation, (b) initiation of rescue therapy, or (c) the end of post-induction maintenance period follow-up or enrollment in a post-maintenance study. In Fortify, the follow-up period extended to 140 days after the last administered study dose (for a total of 68 weeks).[^18^](#_ENREF_18) In U-ENDURE, the follow-up extended to 30 days after the last administered study dose (for a total of 56 weeks).[^15^](#_ENREF_15)

Rescue therapy in each trial consisted of open-label use of the study drug following inadequate response. In FORTIFY, patients could receive open-label risankizumab (in one IV dose of 1200 mg followed by 360 mg [4 x 90 mg SC injection] every 8 weeks thereafter) starting at the Week 16 visit on the basis of increased symptom activity and objective markers of inflammation.[^18^](#_ENREF_18) In U-ENDURE, patients who met the criteria for inadequate response at or after Week 4 could receive open-label upadacitinib 30 mg QD until the end of the follow-up.[^15^](#_ENREF_15)

Censoring at rescue therapy was not considered in the primary analysis due to the potential dependence between uncontrolled disease and subsequent hospitalization risk, and because the focus of the study was to assess the effect of endoscopic remission (as opposed to the impact of a study intervention).

- 1. Association of endoscopic remission and CD-related hospitalizations

A total of 61 distinct CD-related hospitalizations were recorded during the sensitivity analysis exposure period (maintenance baseline to the earliest of (a) study discontinuation, (b) initiation of rescue therapy, or (c) the end of maintenance period follow-up or enrollment in a post-maintenance study. The timing of CD-related hospitalization was relatively uniform across the study period, with approximately 25% of events occurring within the first 41 days, and approximately 75% of events occurring within the first 202 days. Of recorded events, 52 CD-related hospitalizations occurred in the 634 non-remission patients in a cumulative exposure time of 487.9 person-years (0.107 events per person-year). By contrast, among the 235 patients with endoscopic remission, only 9 CD-related hospitalizations were recorded over a total exposure time of 208.4 person-years (0.043 events per person-year). Cumulative CD-related hospitalization events by time from study baseline for each group are presented in

Supplemental Figure 1.

Supplemental Figure 1: Cumulative number of hospitalization events per patient over time stratified by endoscopic remission status in the sensitivity analysis exposure window

- 1. Association of endoscopic response and CD-related hospitalizations

Endoscopic remission status was determined according to the trial methodology for assessing achievement of this endpoint in induction-phase efficacy analyses. Patients were considered in endoscopic remission if they had a decrease in SES-CD > 50% from Baseline of the induction study (or for patients with an SES-CD of 4 at Baseline, at least a 2-point reduction from Baseline), as scored by central reviewer.

Supplemental Table 1 below presents the results for the endoscopic response multivariable negative binomial regression. These results suggest that post-induction endoscopic response is associated with a reduction in CD-related hospitalizations, however, the estimated IRR did not reach statistical significance (IRR = 0.77, 95% confidence interval [CI] [0.43 – 1.39], *p* = 0.385).

Supplemental Table 1 Multivariable negative binomial regression results for endoscopic response and CD-related hospitalization rates

| Parameter | IRR [95% CI] | | Coefficient [SE] | | p-value | |
| --- | --- | --- | --- | --- | --- | --- |
| Endoscopic response | 0.77 [0.43; 1.39] | | -0.260 [0.299] | | 0.385 | |
| Age [+ 5 years] | 0.94 [0.83; 1.06] | | -0.062 [0.063] | | 0.321 | |
| Female | 1.23 [0.71; 2.13] | | 0.205 [0.282] | | 0.467 | |
| Race [vs. White] |  | |  | |  | |
| Asian | 1.87 [0.97; 3.62] | | 0.628 [0.336] | | 0.062 | |
| Other | 1.01 [0.28; 3.72] | | 0.015 [0.663] | | 0.982 | |
| Hispanic [vs. not] | 1.69 [0.61; 4.72] | | 0.525 [0.524] | | 0.316 | |
| Disease location [vs. Colonic] |  | |  | |  | |
| Ileal-only | 2.75 [1.05; 7.20] | | 1.011 [0.491] | | 0.039 | |
| Ileal-colonic | 1.99 [1.09; 3.64] | | 0.688 [0.308] | | 0.025 | |
| Disease duration [years] | 0.99 [0.96; 1.03] | | -0.006 [0.020] | | 0.741 | |
| Pre-induction (baseline) steroid use | 2.55 [1.47; 4.41] | | 0.934 [0.280] | | <.001 | |
| Previous biologic failure | 2.77 [1.25; 6.18] | | 1.021 [0.409] | | 0.012 | |
| CDAI [+10, maintenance-baseline] | 1.03 [0.99; 1.07] | | 0.030 [0.019] | | 0.119 | |
| Post-induction maintenance treatment [vs. Placebo (UPA trial)] ^a^ |  | |  | |  | |
| Placebo (RZB trial) ^a^ | 0.32 [0.13; 0.77] | | -1.140 [0.450] | | 0.011 | |
| Risankizumab 180 mg SC Q8W | 0.19 [0.07; 0.54] | | -1.637 [0.523] | | 0.002 | |
| Risankizumab 360 mg SC Q8W | 0.39 [0.16; 0.98] | | -0.934 [0.468] | | 0.046 | |
| Upadacitinib 15 mg QD | 0.58 [0.25; 1.38] | | -0.539 [0.439] | | 0.220 | |
| Upadacitinib 30 mg QD | 0.45 [0.19; 1.06] | | -0.808 [0.441] | | 0.067 | |
|  | |  | |  | |  |

^a^ These are the treatment arms resulting from the re-randomization before the entry in the 52-week post-induction maintenance studies not to be confounded with the induction studies randomization arms. Patients randomized to placebo for the post-induction maintenance study are presented separately based on the post-induction maintenance Phase III study source (RZB or UPA trial).

Abbreviations: CDAI = Crohn’s Disease Activity Index; CI = Confidence interval; IRR = Incidence rate ratio; Q8W = Once every 8 weeks; QD = Once daily; RZB = Risankizumab; SC = Subcutaneous; SE = Standard error; UPA = Upadacitinib.

- 1. Association of endoscopic response and all-cause hospitalizations

Supplemental Table 2 below presents the results for the endoscopic response multivariable negative binomial regression for the association of post-induction endoscopic response and all-cause hospitalizations. The association between endoscopic response and all-cause hospitalizations is weaker than the association with CD-related hospitalizations. The estimated IRR indicates a marginal but not statistically significant reduction in hospitalizations (IRR = 0.97, 95% CI: [0.64 – 1.46], *p* = 0.867).

Supplemental Table 2 Multivariable negative binomial regression results for endoscopic response and all-cause hospitalization rates

| Parameter | IRR [95% CI] | | Coefficient [SE] | | p-value | |
| --- | --- | --- | --- | --- | --- | --- |
| Endoscopic response | 0.97 [0.64; 1.46] | | -0.036 [0.212] | | 0.867 | |
| Age [+ 5 years] | 1.08 [1.00; 1.17] | | 0.077 [0.040] | | 0.055 | |
| Female | 1.22 [0.82; 1.81] | | 0.200 [0.201] | | 0.319 | |
| Race [vs. White] |  | |  | |  | |
| Asian | 1.75 [1.07; 2.88] | | 0.560 [0.254] | | 0.027 | |
| Other | 1.00 [0.40; 2.52] | | 0.001 [0.472] | | 0.998 | |
| Hispanic [vs. not] | 1.25 [0.56; 2.78] | | 0.226 [0.407] | | 0.579 | |
| Disease location [vs. Colonic] |  | |  | |  | |
| Ileal-only | 1.52 [0.78; 2.94] | | 0.418 [0.337] | | 0.215 | |
| Ileal-colonic | 1.49 [0.97; 2.29] | | 0.401 [0.218] | | 0.065 | |
| Disease duration [years] | 0.99 [0.97; 1.02] | | -0.008 [0.012] | | 0.532 | |
| Pre-induction (baseline) steroid use | 2.04 [1.36; 3.05] | | 0.711 [0.207] | | <.001 | |
| Previous biologic failure | 1.52 [0.93; 2.49] | | 0.421 [0.250] | | 0.092 | |
| CDAI [+10, maintenance-baseline] | 1.04 [1.01; 1.07] | | 0.037 [0.014] | | 0.006 | |
| Post-induction maintenance treatment [vs. Placebo (UPA trial)] ^a^ |  | |  | |  | |
| Placebo (RZB trial) ^a^ | 0.44 [0.23; 0.82] | | -0.827 [0.322] | | 0.010 | |
| Risankizumab 180 mg SC Q8W | 0.42 [0.22; 0.81] | | -0.860 [0.332] | | 0.010 | |
| Risankizumab 360 mg SC Q8W | 0.53 [0.27; 1.01] | | -0.642 [0.335] | | 0.055 | |
| Upadacitinib 15 mg QD | 0.49 [0.25; 0.96] | | -0.711 [0.344] | | 0.039 | |
| Upadacitinib 30 mg QD | 0.57 [0.30; 1.07] | | -0.560 [0.321] | | 0.081 | |
|  | |  | |  | |  |

^a^ These are the treatment arms resulting from the re-randomization before the entry in the 52-week post-induction maintenance studies not to be confounded with the induction studies randomization arms. Patients randomized to placebo for the post-induction maintenance study are presented separately based on the post-induction maintenance Phase III study source (RZB or UPA trial).

Abbreviations: CDAI = Crohn’s Disease Activity Index; CI = Confidence interval; IRR = Incidence rate ratio; Q8W = Once every 8 weeks; QD = Once daily; RZB = Risankizumab; SC = Subcutaneous; SE = Standard error; UPA = Upadacitinib.

- 1. Association of endoscopic remission with CD-related and all-cause hospitalizations using the sensitivity analysis time-at-risk window

As in the primary analysis, patients achieving endoscopic remission at post-induction maintenance baseline experienced lower rates of hospitalization when considering only events occurring prior to initiation of rescue therapy. In 208.4 person-years of exposure, 9 CD-related hospitalizations (0.043 per person-year) and 25 all-cause hospitalizations (0.120 per person-year) were recorded in patients with baseline endoscopic remission. By comparison, over 487.9 person-years, 52 CD-related hospitalizations (0.107 per person-year) and 100 all-cause hospitalizations (0.205 per person-year) were observed in patients failing to achieve remission. Notably, the observed rates of hospitalization (CD-related and all-cause) for patients in both groups were lower compared to those obtained in the primary analysis, suggesting an increased risk of hospitalization after initiation of rescue therapy.

Regression model estimates were similar to those obtained in the primary analysis. Endoscopic remission was associated with a statistically significant reduction in CD-related hospitalization in both univariable (IRR = 0.39, 95% CI [0.16-0.92], *p* = 0.032) and multivariable regressions (IRR = 0.39, 95% CI [0.16-0.96], *p* = 0.040). Endoscopic remission was associated with a statistically significant reduction in all-cause hospitalization in univariable analyses (IRR = 0.59, 95% CI [0.3-0.98], *p* = 0.043), although the effect was not significant at the 5% level when controlling for other factors (IRR = 0.64, 95% CI [0.38-1.08], *p* = 0.095). Complete results are presented in Supplemental Table 3 below.

Supplemental Table 3 Multivariable negative binomial regression results with censoring at rescue therapy

| Parameter | CD-related hospitalization | | | |  | All-cause hospitalization | | | | | |
| --- | --- | --- | --- | --- | --- | --- | --- | --- | --- | --- | --- |
|  | IRR [95% CI] | | *p*-value | |  | IRR [95% CI] | | | | *p*-value | |
| Endoscopic remission | 0.39 [0.16; 0.96] | | 0.040 | |  | 0.64 [0.38; 1.08] | | | | 0.095 | |
| Age [+ 5 years] | 0.97 [0.84; 1.13] | | 0.696 | |  | 1.08 [0.99; 1.18] | | | | 0.079 | |
| Female | 1.55 [0.79; 3.05] | | 0.206 | |  | 1.49 [0.96; 2.32] | | | | 0.076 | |
| Race [vs. White] |  | |  | |  |  | | | |  | |
| Asian | 3.19 [1.45; 6.99] | | 0.004 | |  | 2.17 [1.25; 3.78] | | | | 0.006 | |
| Other | 1.36 [0.31; 6.06] | | 0.685 | |  | 1.22 [0.45; 3.27] | | | | 0.698 | |
| Hispanic [vs. not] | 2.48 [0.79; 7.75] | | 0.119 | |  | 1.53 [0.66; 3.56] | | | | 0.327 | |
| Disease location [vs. Colonic] |  | |  | |  |  | | | |  | |
| Ileal-only | 2.56 [0.80; 8.16] | | 0.112 | |  | 1.41 [0.69; 2.90] | | | | 0.351 | |
| Ileal-colonic | 2.02 [0.97; 4.20] | | 0.061 | |  | 1.45 [0.91; 2.32] | | | | 0.116 | |
| Disease duration [years] | 1.00 [0.96; 1.04] | | 0.967 | |  | 1.00 [0.97; 1.02] | | | | 0.722 | |
| Pre-induction baseline steroid use | 2.42 [1.24; 4.72] | | 0.010 | |  | 2.11 [1.35; 3.32] | | | | 0.001 | |
| Previous biologic failure | 2.45 [0.98; 6.12] | | 0.056 | |  | 1.28 [0.76; 2.16] | | | | 0.352 | |
| CDAI [+10, maintenance-baseline] | 1.03 [0.98; 1.08] | | 0.242 | |  | 1.05 [1.02; 1.08] | | | | 0.002 | |
| Post induction maintenance treatment [vs. Placebo (UPA trial)]^a^ |  | |  | |  |  | | | |  | |
| Placebo (RZB trial) ^a^ | 0.24 [0.07; 0.79] | | 0.019 | |  | 0.41 [0.19; 0.87] | | | | 0.020 | |
| Risankizumab 180 mg SC Q8W | 0.24 [0.07; 0.80] | | 0.020 | |  | 0.47 [0.22; 0.97] | | | | 0.042 | |
| Risankizumab 360 mg SC Q8W | 0.49 [0.16; 1.49] | | 0.210 | |  | 0.65 [0.31; 1.34] | | | | 0.242 | |
| Upadacitinib 15 mg QD | 0.52 [0.16; 1.64] | | 0.262 | |  | 0.50 [0.22; 1.11] | | | | 0.088 | |
| Upadacitinib 30 mg QD | 0.35 [0.11; 1.08] | | 0.067 | |  | 0.48 [0.22; 1.02] | | | | 0.057 | |
|  | |  | |  | | |  |  |  | |  |

^a^ These are the treatment arms resulting from the re-randomization before the entry in the 52-week post-induction maintenance studies not to be confounded with the induction studies randomization arms. Patients randomized to placebo for the post-induction maintenance study are presented separately based on the post-induction maintenance Phase III study source (RZB or UPA trial).

Abbreviations: CDAI = Crohn’s Disease Activity Index; CI = Confidence interval; IRR = Incidence rate ratio; N/A = Not applicable; Q8W = Once every 8 weeks; QD = Once daily; RZB = Risankizumab; SC = Subcutaneous; SE = Standard error; UPA = Upadacitinib.

- 1. Comparison of risankizumab and upadacitinib results from the induction studies and 52-week maintenance studies

Supplemental Table 4 below presents the weighted average Week 12 results for risankizumab and upadacitinib.[[1](#_ENREF_1),[2](#_ENREF_2)] The results presented for CDAI clinical remission, endoscopic remission, and endoscopic remission are comparable when looking at active treatment arms and the placebo arm of each study. However, the placebo Week 52 results from the two maintenance studies differ substantially, as shown in Supplemental Table 5. The placebo arm from the risankizumab FORTIFY Study performs significantly better than the placebo arm from the upadacitinib U-ENDURE Study.

Supplemental Table 4 Weighted average results from the induction studies[[1](#_ENREF_1),[2](#_ENREF_2)]

| **Treatment Arm** | **CDAI Clinical Remission**  **(%)** | **Endoscopic Response**  **(%)** | **Endoscopic Remission**  **(%)** |
| --- | --- | --- | --- |
| Weighted average of risankizumab ADVANCE and MOTIVATE Week-12 results | | | |
| RZB 600mg | 44.02 | 36.05 | 22.39 |
| RZB 1200mg | 41.13 | 32.83 | 22.64 |
| PBO | 22.10 | 11.60 | 6.63 |
| Weighted average of upadacitinib U-EXCEL and U-EXCEED Week 12 results | | | |
| UPA 45 mg | 44.4 | 40.3 | 24.2 |
| PBO | 25.2 | 8.4 | 4.9 |

CDAI: Crohn’s Disease Activity Index; PBO: placebo; UPA: upadacitinib; RZB: risankizumab.

Supplemental Table 5 Week 52 results from the maintenance studies[[1](#_ENREF_1),[3](#_ENREF_3)]

| **Treatment Arm** | **CDAI Clinical Remission** | **Endoscopic Response** | **Endoscopic Remission** |
| --- | --- | --- | --- |
| Risankizumab FORTIFY Study | | | |
| RZB 180mg | 55.41 | 47.13 | 29.94 |
| RZB 360mg | 52.48 | 46.81 | 39.01 |
| PBO | 40.85 | 21.95 | 12.80 |
| Upadacitinib U-ENDURE Study | | | |
| UPA 15 mg | 37.3 | 27.6 | 19.1 |
| UPA 30 mg | 47.6 | 40.1 | 28.6 |
| PBO | 15.1 | 7.3 | 5.5 |

CDAI: Crohn’s Disease Activity Index; PBO: placebo; UPA: upadacitinib; RZB: risankizumab.
